# Human dissection and comparative anatomy: Archaeological evidence for nineteenth‐century anatomical practice at University College London

**DOI:** 10.1111/joa.70220

**Published:** 2026-08-04

**Authors:** Wendy Birch, Tania Kausmally

**Affiliations:** ^1^ Department of Cell and Developmental Biology University College London London UK; ^2^ Institute of Archaeology, University College London London UK

**Keywords:** anatomical education, Anatomy Act 1832, comparative anatomy, dissection, human remains, medical archaeology, nineteenth‐century Britain

## Abstract

The history of anatomical dissection in nineteenth‐century Britain has largely been reconstructed from archival sources, including legislation, institutional records and medical writings. Archaeological evidence for the material practices of anatomy schools, however, remains comparatively limited. In 2010, redevelopment within the main quadrangle of University College London (UCL) revealed a substantial assemblage of human and animal skeletal remains associated with the institution's early anatomy school. Excavation recovered more than 8700 human skeletal fragments and over 800 animal bones, together with artefacts dating to the late nineteenth century. Osteological analysis indicates a minimum of 38 individuals, predominantly adults aged 30–50 years, with an overall male:female ratio of approximately 2:1. Only a small proportion of bones exhibited cut marks consistent with dissection, suggesting that the assemblage partly represents the discard of a curated teaching collection rather than solely the residue of routine student dissection. The associated animal remains, dominated by domesticated taxa including cattle, sheep/goat and horse, indicate that comparative anatomy formed an integral component of the same pedagogical environment. Considered in the context of the Anatomy Act of 1832 and the expansion of metropolitan medical education, the assemblage provides rare material evidence for the procurement, pedagogical use, preparation, curation and eventual disposal of both human and animal anatomical material at one of Britain's earliest secular medical schools. The quadrangle assemblage therefore offers a rare opportunity to reflect on the everyday practice of anatomy and the complex relationship between medical education, comparative anatomy, society and the bodies of the nineteenth‐century poor in London.

## Graphical abstract

Excavation at University College London revealed more than 8700 human skeletal fragments representing at least 38 individuals, alongside over 800 animal remains. Features consistent with dissection, specimen preparation and curation provide evidence for the use of human and animal material in nineteenth‐century anatomical education, offering rare insight into the material practices of an early London medical school.

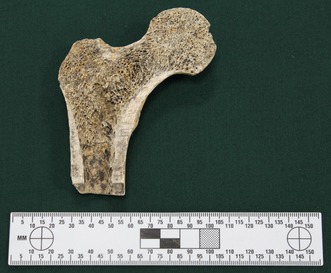

## 1 INTRODUCTION

Human dissection has long occupied a central place in medical education. From the anatomical theatres of early modern Europe to the dissecting laboratories of nineteenth‐century medical schools, the study of the human body through direct anatomical investigation formed the foundation of medical knowledge. However, the history of dissection is not solely one of scientific progress. It is also embedded within wider social, ethical and political debates concerning death, poverty and the control of the body (Hutton, 2013; Mitchell, 2012; Mitchell et al., 2011).

In Britain, the rapid expansion of medical education during the eighteenth and nineteenth centuries created increasing demand for human bodies, generating tensions between scientific need and public attitudes towards the treatment of the dead (Richardson, 1987). Before legislative reform, the legal supply of bodies was limited largely to executed criminals, a source wholly insufficient for the needs of the medical schools. An illicit trade in corpses subsequently developed through the activities of so‐called ‘resurrection men’, who exhumed the dead from burial grounds for sale to anatomists. Public outrage at ‘bodysnatching’, together with several notorious murder cases, ultimately contributed to the passage of the Anatomy Act of 1832, which permitted licensed anatomy schools to receive the unclaimed bodies of individuals who died in workhouses, hospitals and prisons (Hurren, 2012; Richardson, 1987).

Founded in 1826 as a secular alternative to Oxford and Cambridge, University College London quickly established itself as a major centre for medical education in London. The anatomy school, established in the early years of the institution, was formally associated with University College Hospital in 1834 and trained generations of medical students during a period of profound transformation in anatomical teaching (UCL, 2024). UCL also became associated with one of the most unusual figures in the history of anatomy and death: the philosopher Jeremy Bentham (1748–1832). Bentham, whose utilitarian philosophy emphasised rationality and social reform, famously requested that his own body be dissected after death for the benefit of medical science (Southwood Smith, 1832). Following the public dissection of his remains in 1832, Bentham's skeleton was preserved and articulated as the ‘Auto‐Icon’, which remains on display at UCL today (Marmoy, 1958). Bentham's post‐mortem instructions reflect a utilitarian view of the body as a resource for education and scientific advancement and symbolically linked the emerging institution to broader debates surrounding the ethical use of human remains in medicine.

Although historical scholarship has explored the legislative, social and ethical dimensions of anatomical dissection in considerable depth, relatively little is known about the material traces of anatomy schools themselves. Archaeological investigations associated with nineteenth‐century medical institutions, including the Royal London Hospital, Worcester Royal Infirmary, Bristol Royal Infirmary and Newcastle Infirmary, have begun to provide osteological evidence of dissection practices, acquisition of human bodies for anatomical study, specimen preparation and the treatment of anatomical remains (Chamberlain, 2012; Fowler & Powers, 2012; Western & Kausmally, 2010; Witkin, 2011). Such assemblages offer an important counterpoint to documentary records by revealing how anatomical knowledge was produced in practice rather than merely described in written sources.

In early spring 2010, redevelopment work in the south‐west corner of the main UCL quadrangle revealed a substantial deposit of human and animal skeletal remains associated with the early anatomy school. The presence of both human and animal material reflects the dual foundations of nineteenth‐century anatomical education, in which human dissection was complemented by comparative study across a range of animal species. The UCL assemblage therefore provides a rare archaeological window into the practical realities of anatomical education at UCL during the nineteenth century.

This paper examines three principal questions arising from the UCL assemblage: how human bodies used in anatomical education were procured, used and ultimately disposed of; what the presence of non‐adult remains may reveal; and how animal material contributed to the practice of comparative anatomy at UCL. By combining osteological analysis with historical context, this study assesses the demographic composition of the human remains, the evidence for dissection and specimen preparation and the role of animals within nineteenth‐century anatomical education. The aims of this study are to investigate the demographic composition, patterns of modification and archaeological context of the UCL quadrangle assemblage to better understand the acquisition, use, curation and disposal of human and animal anatomical material within nineteenth‐century medical education.

Specifically, this study examines:

- The demographic characteristics of the human remains;
- Evidence for dissection, specimen preparation and curation; and
- The role of animal material in anatomical teaching.

## 2 MATERIALS AND METHODS

### 2.1 Archaeological context

**FIGURE 1:**
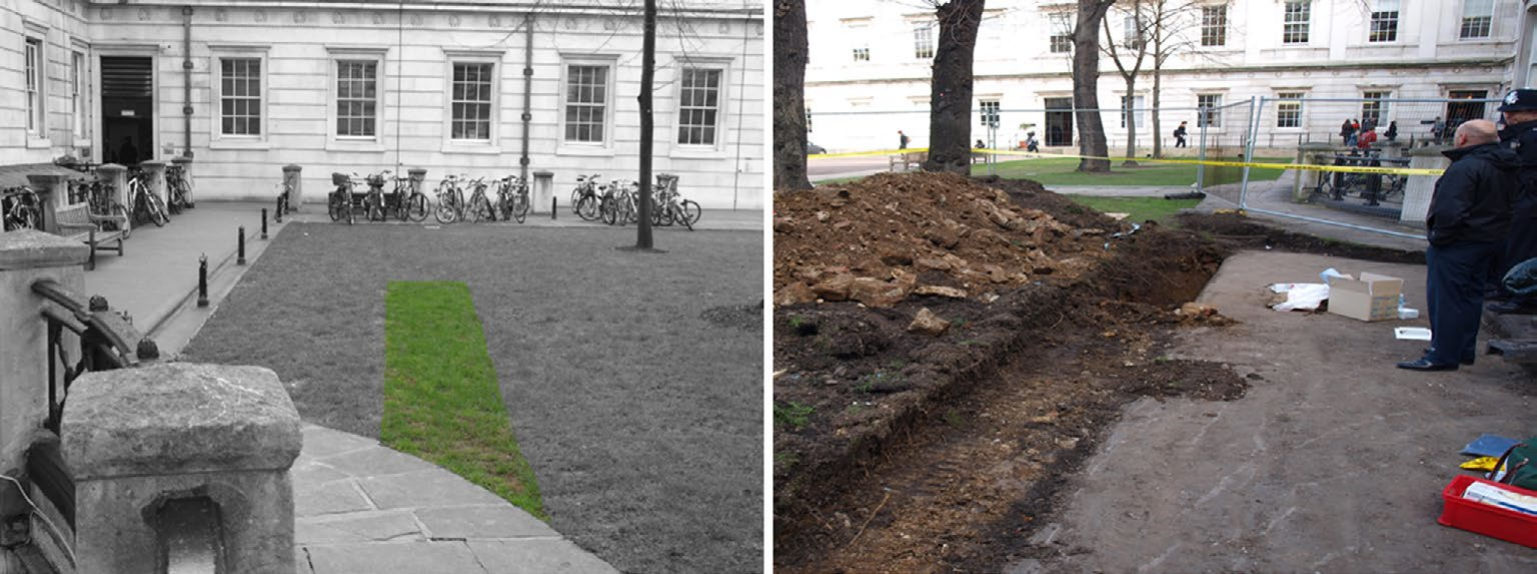
Location of the area where skeletal remains were discovered in the UCL Front Quadrangle in 2010.

Following the discovery of the skeletal remains, a rescue excavation was undertaken by the UCL Department of Cell and Developmental Biology in collaboration with students from the Institute of Archaeology. The excavation revealed a large assemblage of disarticulated skeletal material, some of which exhibited modifications consistent with anatomical dissection. The excavation limits were defined by the redevelopment footprint rather than the full extent of the archaeological deposit (see Figure 1).

The skeletal material is most plausibly derived from the UCL anatomy school and was subsequently discarded. Due to the absence of articulated or partially articulated remains, it was not possible to establish the deposit as a primary deposition. The disarticulated nature of the remains is consistent with the interment of already disarticulated material. Dating evidence recovered from the site included an early hand‐blown 1 oz glass Bovril jar manufactured after 1889, indicating that deposition could not have occurred before this date (Grace's Guide, 2025; Helliwell, 1995). Although deposition occurred sometime after 1889, the remains may be considerably older and may have derived from an anatomical teaching collection or from dissection material used at the anatomy school any time between the school's establishment in 1826 and their eventual disposal in the late nineteenth century.

The excavation yielded more than 8700 human skeletal fragments, over 800 animal skeletal remains and more than 220 associated artefacts.

### 2.2 Osteological analysis of human remains

To characterise the nature and origin of the assemblage, the human skeletal material was examined using standard osteological methods to assess demographic characteristics, pathological conditions and evidence of post‐mortem modification associated with anatomical dissection (Aufderheide & Rodríguez‐Martin, 1998; Buikstra & Ubelaker, 1994; White et al., 2012). The minimum number of individuals (MNI) was estimated from the most frequently represented skeletal elements. Age estimation was based on skeletal fusion and dental development in non‐adults, and on degenerative changes of the pubic symphysis and auricular surface of the os coxa in adults. Biological sex estimation was based primarily on sexually dimorphic features of the os coxa and skull, where preservation permitted.

Pathological changes were recorded macroscopically and classified into major categories, including infection, degeneration, trauma, metabolic disease, congenital variation and miscellaneous lesions. Post‐mortem modifications, including knife, saw and chop marks, were recorded by anatomical region and described according to morphology and probable mechanism. Ink or pencil markings and evidence of bone whitening or preparation were also noted (Mitchell et al., 2011; Powers, 2012).

### 2.3 Zooarchaeological analysis of animal remains

Animal remains were identified to taxon where possible using comparative collections and standard zooarchaeological criteria (Baker & Worley, 2019; Harland et al., 2003). The number of identified specimens (NISP) and MNI were recorded, and age at death was estimated from skeletal fusion where preservation allowed. Anatomical distribution and evidence of modification, including cut marks, sectioning, drilling and surface treatment, were documented to assess whether the assemblage was more consistent with butchery, domestic refuse or anatomical teaching material.

### 2.4 Interpretative framework

When interpreting archaeological material within a historical context, it is important to recognise that archaeological assemblages represent a fundamentally different evidential framework from documentary sources. The skeletal material recovered from the quadrangle was deliberately discarded rather than intentionally curated or archived. In contrast to written records, which usually preserve what contemporaries regarded as important or worthy of recording, the archaeological assemblage reflects material that was no longer regarded as useful by those responsible for its disposal. The analytical questions posed by this material therefore differ from those typically applied to written sources. Rather than revealing what was considered valuable within the anatomy school, the assemblage may instead illuminate material that had become surplus, redundant or pedagogically obsolete, thereby providing insight into the practical realities of anatomical teaching and the life cycle of anatomical specimens within the nineteenth‐century medical school. Throughout this paper, the terms ‘human remains’, ‘bodies’ and ‘anatomical material’ are used in preference to ‘cadavers’ to reflect contemporary professional and ethical terminology while recognising the historical context of nineteenth‐century anatomical practice.

## 3 RESULTS

### 3.1 Human remains

#### 3.1.1 Anatomical region distribution

**FIGURE 2:**
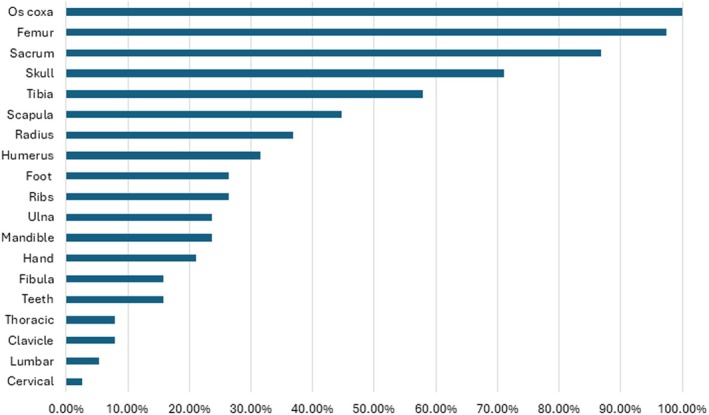
Anatomical region distribution of the human remains based on the minimum number of elements within each anatomical region (MNI = 38).

The remains were highly fragmented and entirely disarticulated, and no complete or partially articulated skeletons were identified. From the more than 8700 human skeletal fragments, it was possible to identify the presence of at least 38 individuals, based on the most frequently occurring element in the assemblage, the os coxa (see Figure 2). The frequency of different anatomical regions varied markedly. Pelvic elements, followed by femora and sacra, together with cranial fragments, were particularly well represented. By contrast, vertebral elements were notably scarce.

#### 3.1.2 Demographic profile

Age estimation based on skeletal fusion and dental development produced an approximate adult‐to‐child ratio of 4:1. In late‐fusing elements such as the clavicle (fusion completes at 16–21 years) and the iliac crest (fusion completes at 21–23 years), fusion rates were lower, suggesting that some elements classified as adult may derive from late adolescents or young adults. A similar pattern was observed in the dentition, where the majority of developing teeth belonged to individuals aged approximately 12–17 years.

Several age groups could be identified within the non‐adult assemblage. Although no intrauterine remains or individuals aged 7–11 months were identified, the assemblage included individuals aged 1–6 months (8%), 1–5 years (17%), 6–11 years (33%) and 12–17 years (42%). This pattern suggests an increase in representation with age; however, the observed distribution may not reflect the true age profile of the non‐adult population, as intrauterine and neonatal remains are particularly difficult to recover without systematic sieving, and they may also be disproportionately affected by taphonomic loss due to their fragility.

Adult age estimation was based on the os coxa, particularly degenerative changes of the pubic symphysis (*n* = 18) and auricular surface (*n* = 51). These analyses showed that the largest cohort fell within the middle‐aged group of 30–50 years, accounting for approximately 51%–56% of individuals. Younger adults aged 18–30 years accounted for approximately 22%–27%, while individuals aged over 50 represented approximately 22%. Overall, the assemblage appears to comprise approximately 80% adults and 20% non‐adults, although some late‐adolescent elements were likely included within the adult category.

The overall male:female ratio, based on the most reliable sexually dimorphic features, principally the skull and os coxa, was approximately 2:1. When sex estimates were considered by adult age category, males predominated in the younger (64%) and older (81%) age groups, whereas females were slightly more represented within the middle‐aged cohort (58%).

#### 3.1.3 Pathological profile

The prevalence of pathology and trauma within the assemblage was low, although this may partly reflect the high degree of fragmentation. Furthermore, identifying disease in disarticulated remains is inherently difficult, as diagnosis often depends on lesion distribution across multiple skeletal elements. Approximately 3% of the assemblage exhibited bone or dental pathological changes.

Periosteal new bone formation represented the most frequently observed pathological change (*n* = 130). Although such lesions may occur in association with infection, they are non‐specific and may also result from trauma, metabolic disorders, vascular conditions or other systemic disease processes (Weston, 2008). Most lesions consisted of mild active or healed periosteal reactions affecting the lower limb elements. A small number of fragments exhibited lesions more strongly suggestive of infectious processes, including changes consistent with osteomyelitis or infective osteitis. Consequently, although infectious disease was likely present within the assemblage, its prevalence cannot be determined reliably from periosteal new bone formation alone.

Degenerative joint disease was the second most frequent condition (*n* = 67). Degenerative changes were observed primarily in the spine, ribs and os coxa and were typically expressed as mild to moderate marginal osteophytic lipping, with only a limited number of elements exhibiting eburnation sufficient to support a diagnosis of osteoarthritis.

Dental pathology (*n* = 37) included caries, calculus, enamel hypoplasia, periodontitis and periapical lesions. Enamel hypoplasia was present in five individuals, indicating episodes of chronic physiological stress during childhood. Partial tooth loss was recorded in seven individuals, while carious lesions were observed in five.

Trauma (*n* = 30) was represented predominantly by healed rib fractures. Other lesions included avulsion fractures of the proximal humerus and blunt‐force trauma to the glabella region of the frontal bone.

Metabolic conditions were less frequent (*n* = 13). Most consisted of *cribra orbitalia*, which manifests as porous lesions on the roof of the orbit and is believed to be vascular in nature. Although the aetiology is unknown, it is thought to be a non‐specific indicator of metabolic stress commonly linked, though not exclusively, to iron deficiency anaemia (Roberts & Manchester, 2010). Other metabolic lesions included evidence of healed rickets, which had affected the lower limbs and which indicates vitamin D deficiency during childhood.

Congenital conditions (*n* = 10) included skeletal variations primarily affecting sacral and tibial elements, and including a bifid spinous process. A single circulatory condition was identified in the form of *osteochondritis dissecans* on a femoral lateral condyle. Other miscellaneous conditions included a case of *hyperostosis frontalis interna* (*HFI*), a condition characterised by thickening of the internal surface of the frontal bone. This is usually a benign condition predominantly found in mature females (Aufderheide & Rodríguez‐Martin, 1998). One possible case of Paget's disease was also observed. Paget's is a slowly progressive disorder that disrupts the normal cycle of bone growth, leading to significant thickening and bending of the bone due to its weakened state. It usually occurs in elderly individuals (Adams & Hamblen, 2001).

#### 3.1.4 Post‐mortem modification

Only a small proportion of human skeletal fragments exhibited post‐mortem modification in the form of knife, saw or chop marks. Of the elements identified to anatomical region, approximately 3% showed clear evidence of modification. Knife marks were the most common, accounting for 86% of all modifications. Saw marks accounted for 12% and chop marks for 2%.

**FIGURE 3:**
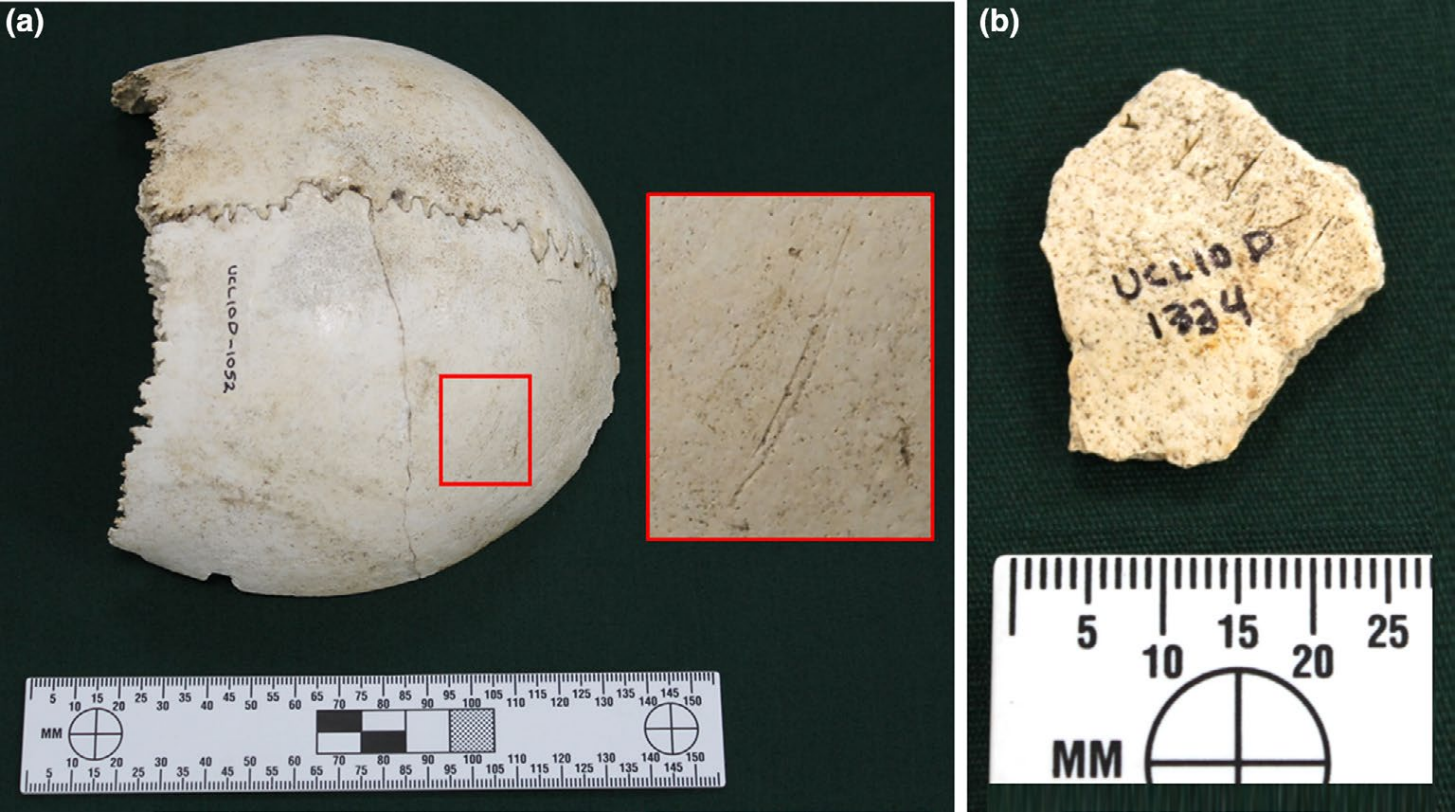
Two examples of the fine parallel striations observed on the human cranial bones, consistent with a blade passing across the cortical bone surface during soft‐tissue removal.

**FIGURE 4:**
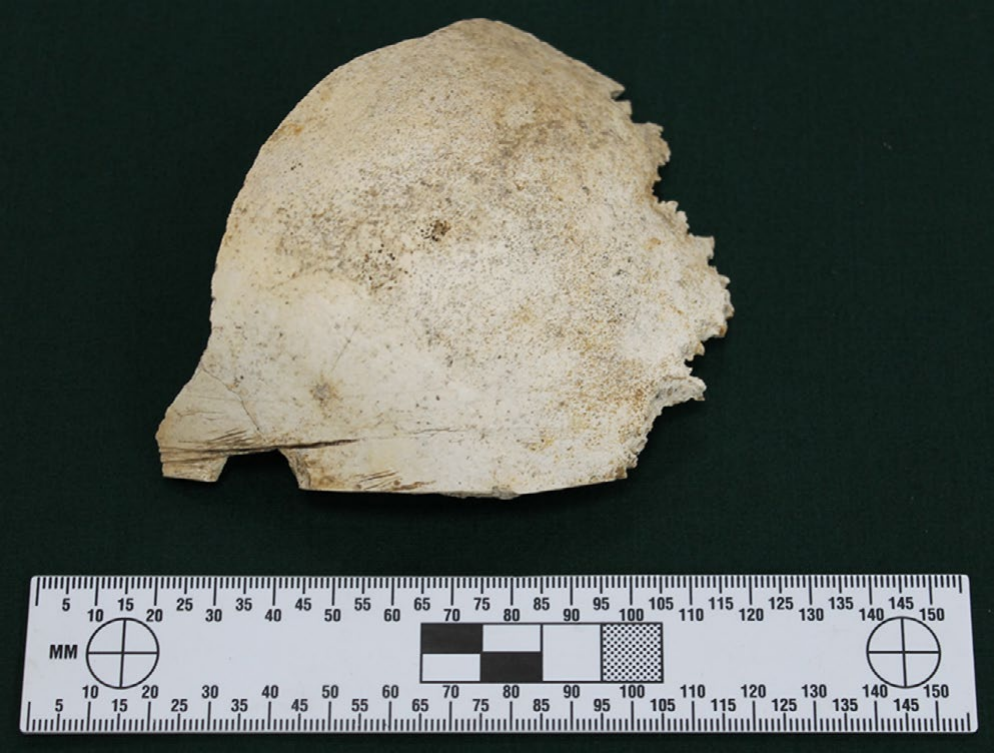
Example of the saw marks observed on the human cranial bones, consistent with calvarium removal.

**FIGURE 5:**
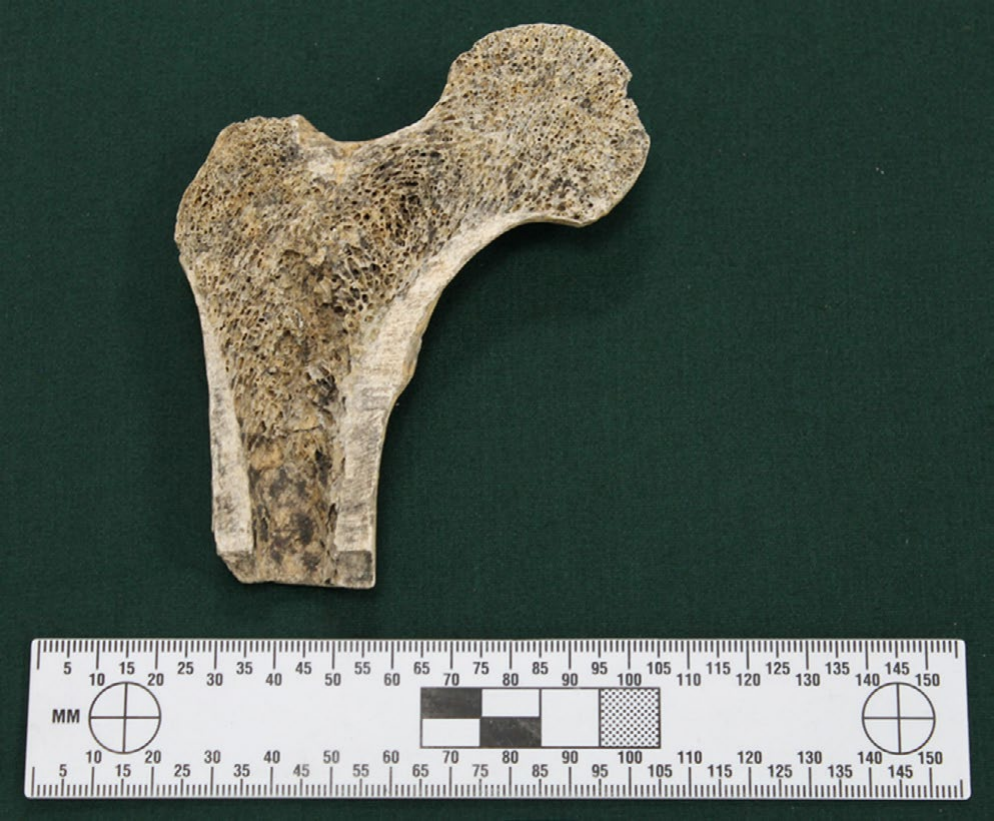
Example of the saw marks observed on the human femur, consistent with longitudinal sectioning to expose the internal structure of the bone.

**FIGURE 6:**
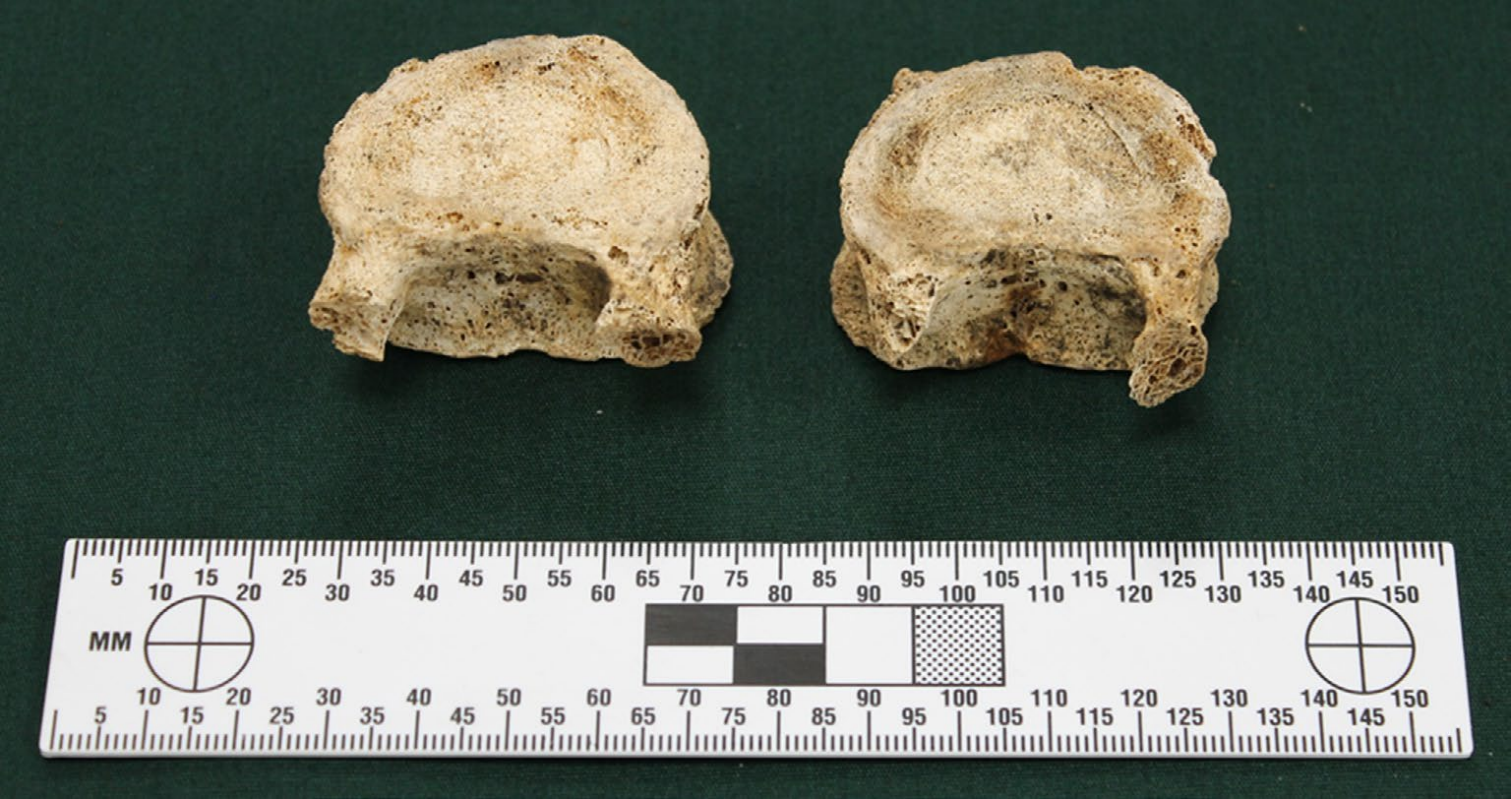
Example of the saw marks on two human lumbar vertebrae which may indicate removal of the vertebral arch to expose the spinal cord in situ.

Knife marks were predominantly observed on cranial fragments, long bones and pelvic elements. They frequently appeared as fine parallel striations, consistent with a blade passing across the cortical bone surface during soft‐tissue removal (see Figure 3). Saw marks were present on cranial fragments, particularly the frontal, parietal and occipital bones, and likely represent craniotomy procedures associated with the removal of the calvarium (see Figure 4). Saw marks on long bones may reflect the division of bodies for teaching purposes and longitudinal sectioning of long bones to demonstrate their internal structure (see Figure 5), while saw marks on lumbar vertebrae may indicate removal of the vertebral arch to expose the spinal cord in situ (see Figure 6). Chop marks were rare, occurring on only four fragments, including femora, a rib and a parietal bone.

**FIGURE 7:**
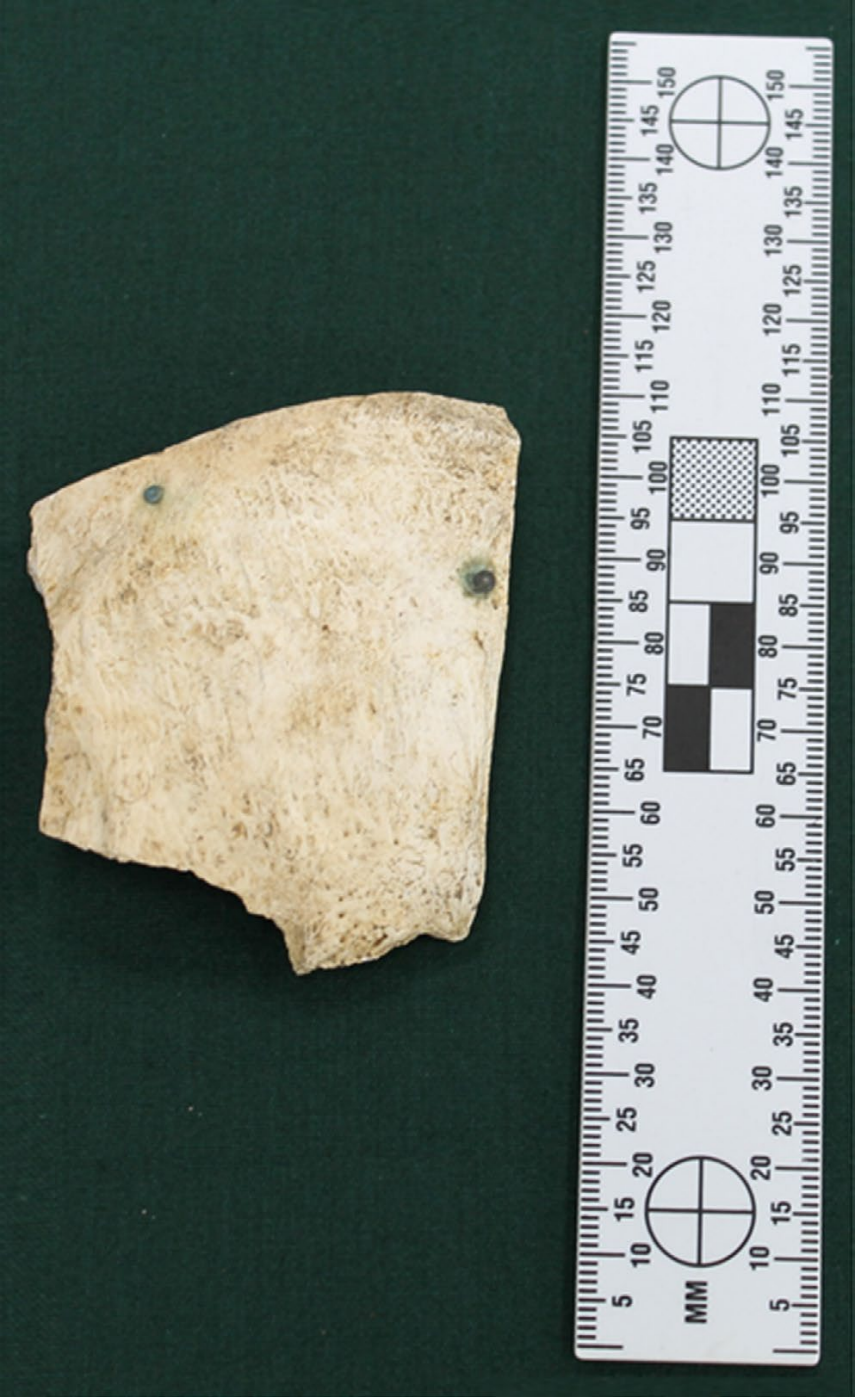
Sectioned human occipital bone exhibiting metal fittings for attachment to adjacent cranial components, consistent with preparation as a teaching specimen.

One sectioned occipital bone fragment exhibited metal fittings, indicating attachment to other cranial components, consistent with its use as a teaching specimen (see Figure 7). Markings were recorded on 10 fragments in the form of numbers or letters applied in ink or pencil, suggesting that some of the bones had been handled in a dry state. Approximately 20% of the identified fragments exhibited a distinctive ‘white’ colouration, with 84% of the affected elements belonging to cranial bones, consistent with chemical treatment or whitening.

### 3.2 Animal bones

#### 3.2.1 Assemblage composition and species distribution

**FIGURE 8:**
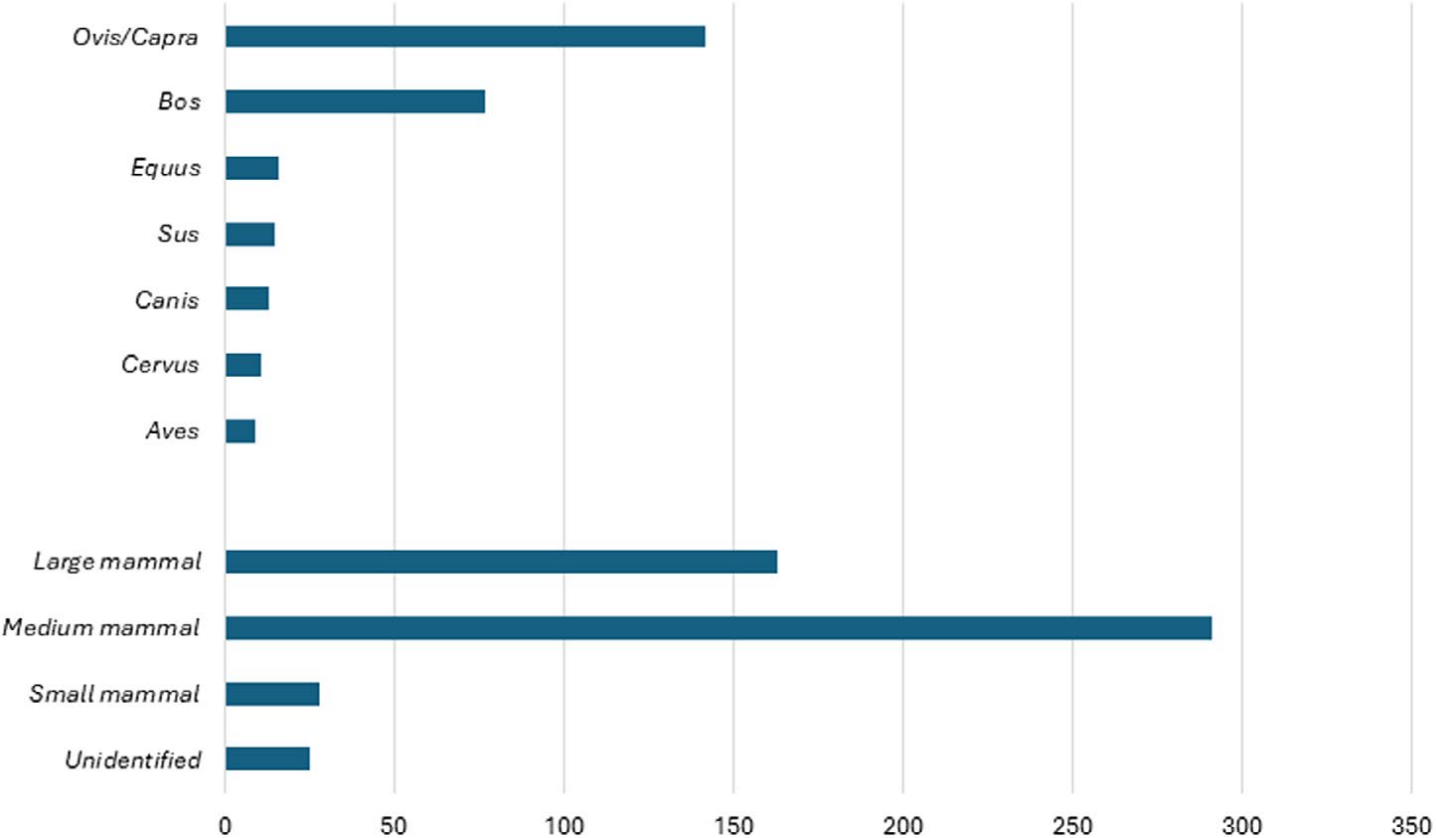
Species distribution of animal remains based on the number of identified specimens (NISP = 805).

Animal remains constituted approximately 8% of the total skeletal material recovered from the UCL quadrangle. More than 800 animal bone fragments were identified. The species composition was dominated by medium‐ and large‐sized domesticated animals (see Figure 8). The most common taxa, based on NISP, were sheep/goat, followed by cattle and horse. Other species present included pig, dog, cat, deer, rabbit/hare and various birds.

**FIGURE 9:**
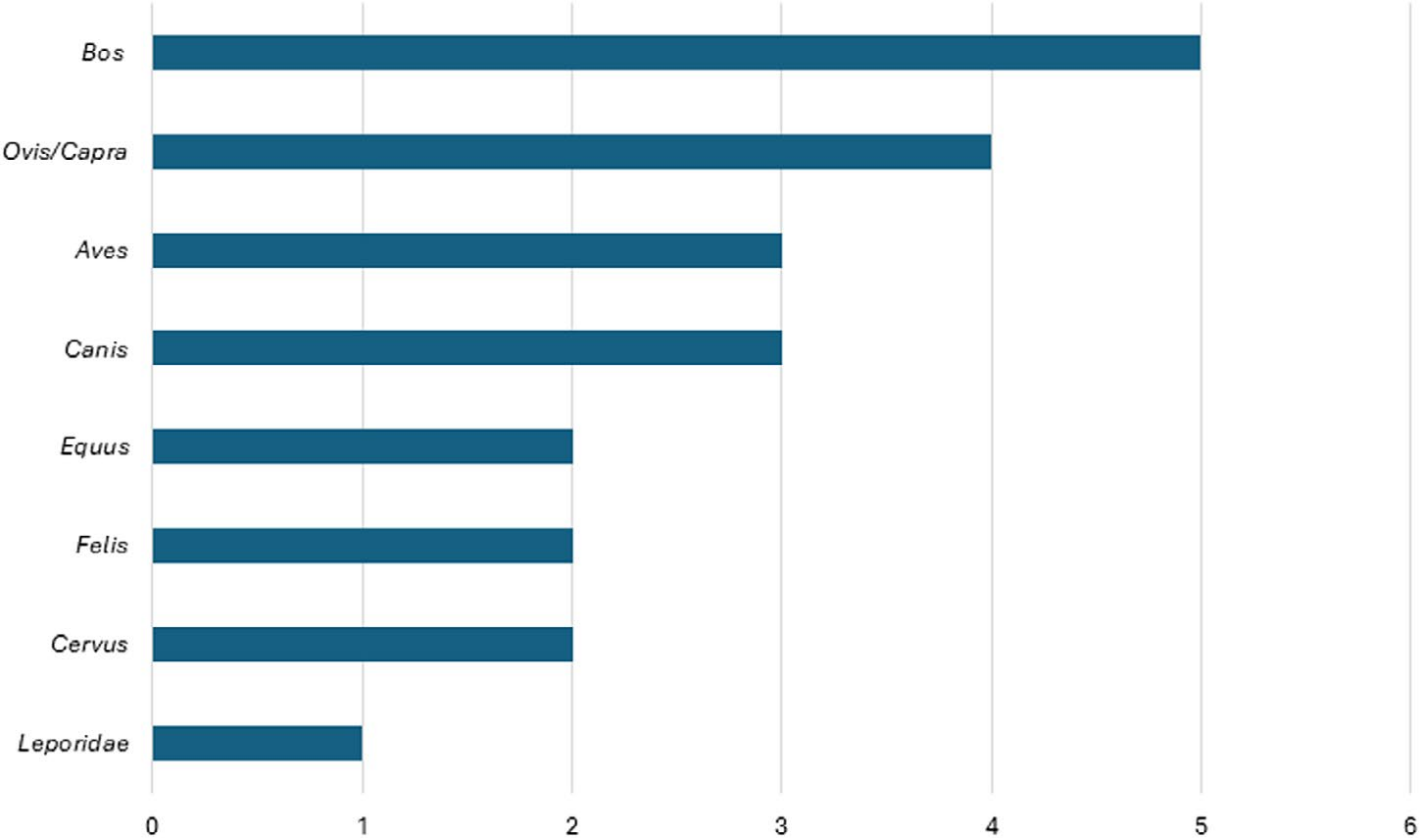
Distribution of animal remains by minimum number of individuals (MNI = 22).

Analysis of MNI suggested that at least 22 individual animals were represented within the UCL assemblage (see Figure 9). Based on MNI, cattle were the most common species, followed by sheep/goat, birds, dog, horse, cat and deer, with a single rabbit/hare identified. This distribution differs somewhat from the pattern suggested by fragment counts alone. These differences highlight the need for caution when interpreting the apparent dominance of particular species within the assemblage. Birds, in particular, are represented by several species, including domestic fowl, goose and swan, which inflates the MNI relative to fragment count.

#### 3.2.2 Anatomical and age distribution

Analysis of anatomical distribution was complicated by the fact that many skeletal elements, particularly ribs and vertebrae, could not be reliably assigned to species and many fragments were too small to preserve diagnostic features. No clear pattern of preferential disposal of particular anatomical regions was observed. Within the species represented by larger numbers of fragments, skeletal elements appeared to be relatively evenly distributed across anatomical regions.

The majority of animal remains exhibited complete skeletal fusion and therefore derived from mature individuals. Evidence for juvenile animals was limited and observed only among sheep/goat and pig remains. The two deer, represented by 11 fragments, suggested the presence of at least one fallow deer. One deer was aged at less than 3–4 years, and the other was older than 3–5 years. Overall, however, the assemblage appears to be dominated by adult animals.

#### 3.2.3 Post‐mortem modification

Patterns of animal post‐mortem modification varied by taxon and indicate a combination of butchery, dissection and specimen preparation. Cut marks were observed on cattle, sheep/goat, pig and deer remains, with the highest frequency relative to fragment count occurring on cattle elements (33%). Many resembled typical butchery marks, although some of the finer marks may have resulted from anatomical dissection.

**FIGURE 10:**
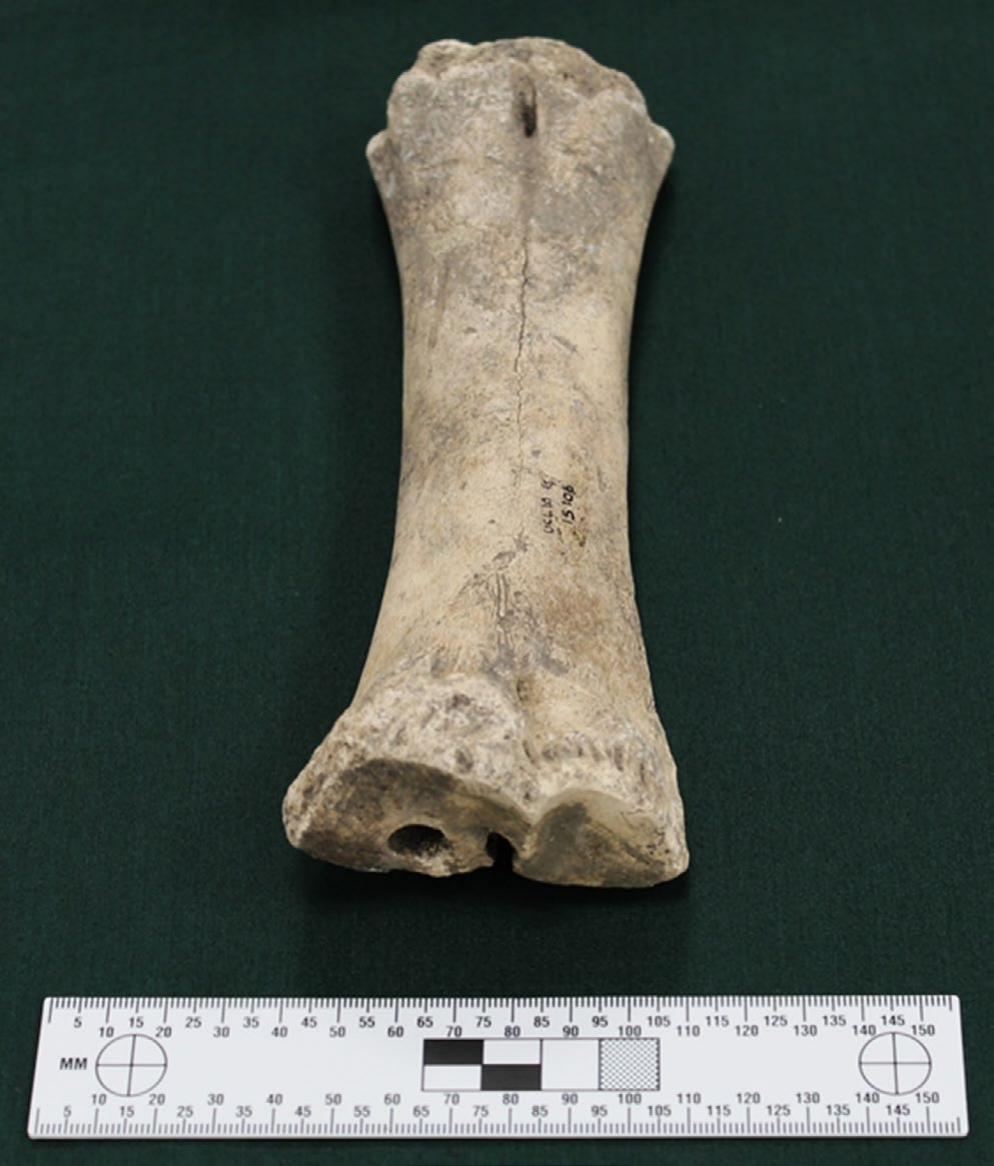
Example of a drill hole in a cattle (*Bos taurus*) metacarpal, indicating deliberate modification consistent with preparation for use as a teaching specimen.

A cattle metacarpal displayed a drilled perforation on the proximal epiphysis (see Figure 10), while another had been sectioned longitudinally. Among the horse remains (*n* = 16), no clear butchery marks were observed despite the animal's large body size, suggesting that horse specimens may have been acquired through different channels than cattle material.

**FIGURE 11:**
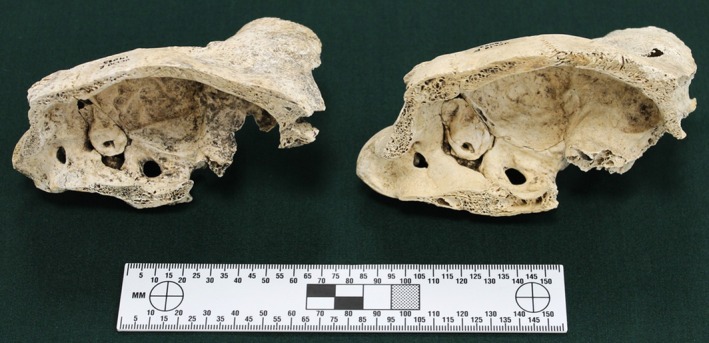
Two examples of sagittally sectioned sheep (*Ovis aries*) skulls, indicating deliberate modification consistent with preparation for use as teaching specimens.

Sheep/goat remains exhibited comparatively fewer butchery marks than those of the larger mammals (5%), and three sheep skull fragments displayed sagittal cuts more consistent with anatomical dissection than butchery, together with evidence of chemical treatment indicative of preparation for demonstration or display (see Figure 11). Pig remains, by contrast, displayed cut marks on approximately one third of fragments, most consistent with butchery practices, while a boar tusk had been sectioned to expose its internal structure. Several cat and dog skeletal elements also exhibited ivory‐white colouration indicative of preparation for anatomical display.

Although the majority of the modifications appear consistent with butchery or dissection, it remains possible that some of the animal material could represent food waste. While none of the bones showed evidence of cooking, two displayed animal gnawing, including a deer tibia, suggesting that rodents had access to the remains prior to burial, either at the anatomy school or through exposure prior to burial. Overall, these patterns suggest a mixed deposit in which non‐human anatomical teaching material was discarded alongside remains deriving from butchery and possibly more general refuse.

## 4 DISCUSSION

### 4.1 Archaeological context and interpretative limitations

The human and animal remains recovered from the UCL quadrangle constitute a rare archaeological assemblage documenting the material realities of anatomical education in nineteenth‐century London. Although the legislative and social contexts of human dissection before and after the Anatomy Act of 1832 have been extensively explored in historical scholarship, physical evidence for the everyday practices of anatomy schools remains comparatively scarce. Even less frequently preserved are the material traces of comparative anatomy, despite its role within nineteenth‐century curricula. The UCL assemblage therefore provides an important opportunity to examine how both human and animal material were integrated within anatomical teaching, how human remains and specimens were used and curated, and how anatomical material was ultimately treated once its perceived pedagogical value had ended. The assemblage is best interpreted as a composite deposit incorporating both routine dissection material and elements derived from curated collections.

Assemblages derived from anatomy schools differ fundamentally from both conventional archaeological burial populations and from faunal assemblages associated with subsistence activity. Rather than representing populations interred through funerary practices or patterns of consumption, such collections reflect institutional processes including body acquisition, the procurement of animal specimens, pedagogical use, specimen preparation, selective retention and eventual disposal. Interpretation of the UCL assemblage must therefore recognise that its composition was shaped by anatomical teaching practices and curricular needs, rather than by natural mortality patterns or dietary behaviour within a wider population. Nevertheless, the assemblage provides evidence of the types of individuals whose bodies entered the anatomy school during the nineteenth century and of the ways in which this anatomical material was used, curated and ultimately discarded.

The disarticulated and highly fragmented nature of the human and animal remains presents considerable analytical challenges. The complete absence of articulated skeletons limits the reconstruction of skeletal associations and complicates demographic, taxonomic and pathological interpretation. Fragmentation limits the association of skeletal elements, reduces the reliability of demographic attribution and complicates the identification of pathology and modification; interpretations must therefore be made at the assemblage level. These limitations are characteristic of anatomy‐school deposits, where both human bodies and animal specimens were subjected to repeated manipulation through dissection, demonstration, specimen preparation and eventual disposal (Fowler & Powers, 2012; Western & Kausmally, 2010). Interpretations of the source of anatomical material, specimen use and pedagogical context must therefore remain probabilistic, since the assemblage represents the eventual disposal of anatomical material and lacks direct documentary association with individual remains or specimens.

### 4.2 Procurement of anatomical material and demographic composition

The demographic profile of the quadrangle assemblage provides important insight into the social origins of the individuals whose bodies were used in anatomical teaching at UCL. The predominance of adults aged 30–50 years, perhaps unsurprisingly, contrasts with the older age profile of body donors at UCL today (typically over 60 years of age). This younger adult age profile is consistent with nineteenth‐century institutional populations, which are widely associated with the urban poor, including labourers, transient individuals and those residing in workhouses, hospitals and prisons, where a combination of factors, including occupational risk, infectious disease exposure, malnutrition and overcrowded living conditions contributed to elevated mortality in early and middle adulthood (Mooney et al., 1999). This age distribution is consistent with the sources of anatomical material established under the Anatomy Act of 1832, whereby unclaimed bodies from such institutions formed the primary supply for anatomical teaching.

Skeletal evidence of pathological change within the assemblage supports this interpretation. Although only approximately 3% of skeletal elements displayed bone or dental pathological changes, the pathological profile nevertheless provides important insight into the health conditions of individuals whose bodies entered nineteenth‐century anatomy schools. Where present, the pathological profile is consistent with patterns widely documented in post‐medieval urban populations and is consistent with the socio‐economic backgrounds of many individuals who died in workhouses and hospitals in the nineteenth century (Roberts & Cox, 2003). Evidence of infectious and chronic disease, alongside indicators of physiological stress, suggests prolonged exposure to conditions associated with poverty, including malnutrition, recurrent infection and sustained physical labour. The presence of lesions suggestive of infection aligns with expectations for densely populated urban environments characterised by overcrowding, poor sanitation and restricted access to healthcare (Roberts & Cox, 2003), while degenerative joint changes reflect cumulative mechanical stress in physically demanding occupations (Roberts & Manchester, 2010).

Dental and metabolic indicators reinforce this interpretation. Enamel hypoplasia indicates episodes of childhood physiological stress, potentially associated with disease, nutritional or weaning‐related stress or other metabolic disturbance (Goodman & Rose, 1990), while patterns of antemortem tooth loss and caries are typical of post‐medieval populations experiencing long‐term dietary and health stress (Mant & Roberts, 2015; Whittaker & Molleson, 1996). Metabolic lesions, primarily *cribra orbitalia*, together with evidence of healed rickets, further point to chronic nutritional deficiency and disease during growth (Brickley & Ives, 2006; Roberts & Manchester, 2010).

Overall, the pathological profile is characteristic of economically marginalised urban populations and closely parallels findings from comparable assemblages (Roberts & Cox, 2003). It therefore reinforces the interpretation that the individuals represented in the UCL assemblage were drawn largely from poor populations, and that the assemblage reflects not only mortality structure but also the cumulative, embodied effects of poverty and disease in nineteenth‐century London.

This demographic and pathological profile is consistent with the mechanisms through which human remains were acquired for anatomical teaching following the Anatomy Act of 1832. By granting anatomy schools access to the unclaimed dead from workhouses, hospitals and prisons, the Act effectively redirected the bodies of the poor into medical education, embedding anatomical practice within existing structures of social inequality (Richardson, 1987). The UCL assemblage can therefore be interpreted as a product of these institutional systems, rather than a reflection of the wider population. Although the specific institutional origin of individual bodies cannot be reconstructed, the demographic and pathological profile is most consistent with acquisition through the institutional systems formalised after 1832, including the workhouse system.

The estimated male:female ratio of approximately 2:1 broadly aligns with patterns observed in other nineteenth‐century anatomical assemblages, although it is somewhat less skewed than the ratios approaching 3:1 reported from hospital‐based sites such as the Royal London Hospital and Worcester Royal Infirmary (Fowler & Powers, 2012; Western & Kausmally, 2010). This imbalance has been attributed to the over‐representation of men among unclaimed institutional deaths, particularly within prison populations and transient labour markets, increasing the likelihood that their bodies would remain unclaimed and therefore be available for dissection (Dittmar & Mitchell, 2018; Hurren, 2012; Richardson, 2001). This pattern may also reflect selection practices as male bodies were often considered more appropriate for anatomical demonstration due to their more visibly defined musculature, whereas the dissection of female bodies may have been shaped by concerns surrounding modesty and propriety and regarded as socially indecorous (Richardson, 2001).

The comparatively more balanced sex ratio at UCL suggests variation in anatomical supply networks between anatomy schools. Many excavated nineteenth‐century anatomy schools in the United Kingdom are associated with hospitals or infirmaries, including the Royal London Hospital (Fowler & Powers, 2012), Worcester Royal Infirmary (Western & Kausmally, 2010), Newcastle Royal Infirmary (Chamberlain, 2012), Bristol Royal Infirmary (Witkin, 2011) and the Radcliffe Infirmary (Loe et al., 2021). These institutions had relatively direct access to human bodies through hospital mortality and therefore often reflect demographic profiles characterised by higher proportions of men, as well as increased representation of both very young and elderly individuals (Waddington, 2000).

Although the UCL anatomy school became associated with University College Hospital (then the North London Hospital) after 1834, the demographic profile of the quadrangle assemblage suggests that it may not have relied on hospital mortality to the same extent as other anatomy schools. The demographic profile may be consistent with a greater reliance on bodies acquired through the workhouse system, which often displayed more balanced sex ratios than hospital or prison populations and frequently included significant numbers of older individuals, particularly women, especially in large urban centres such as London (Green & Owens, 2017; Waddington, 2000). If workhouses constituted a significant source of anatomical material for the UCL anatomy school, this may partly account for the reduced male predominance observed. Differences in anatomical material supply networks may provide one explanation for the demographic variation between the UCL assemblage and other excavated anatomy school populations.

Overall, these patterns indicate that the composition of the UCL assemblage was shaped by anatomical material supply networks and institutional pathways of acquisition, reflecting the intersection of poverty, disease and post‐mortem governance in nineteenth‐century London. The assemblage therefore provides not only evidence for anatomical practice, but also a material record of how socially marginalised populations were incorporated into nineteenth‐century medical education.

### 4.3 The significance of non‐adult remains

The presence of non‐adult remains is a significant feature of the assemblage and demonstrates that child bodies formed part of anatomical teaching at UCL. Although adults predominate, individuals spanning infancy to adolescence are represented, providing rare archaeological evidence for the inclusion of non‐adult material within anatomy schools, which is less clearly documented in historical sources that tend to emphasise adult bodies used for anatomical teaching.

**FIGURE 12:**
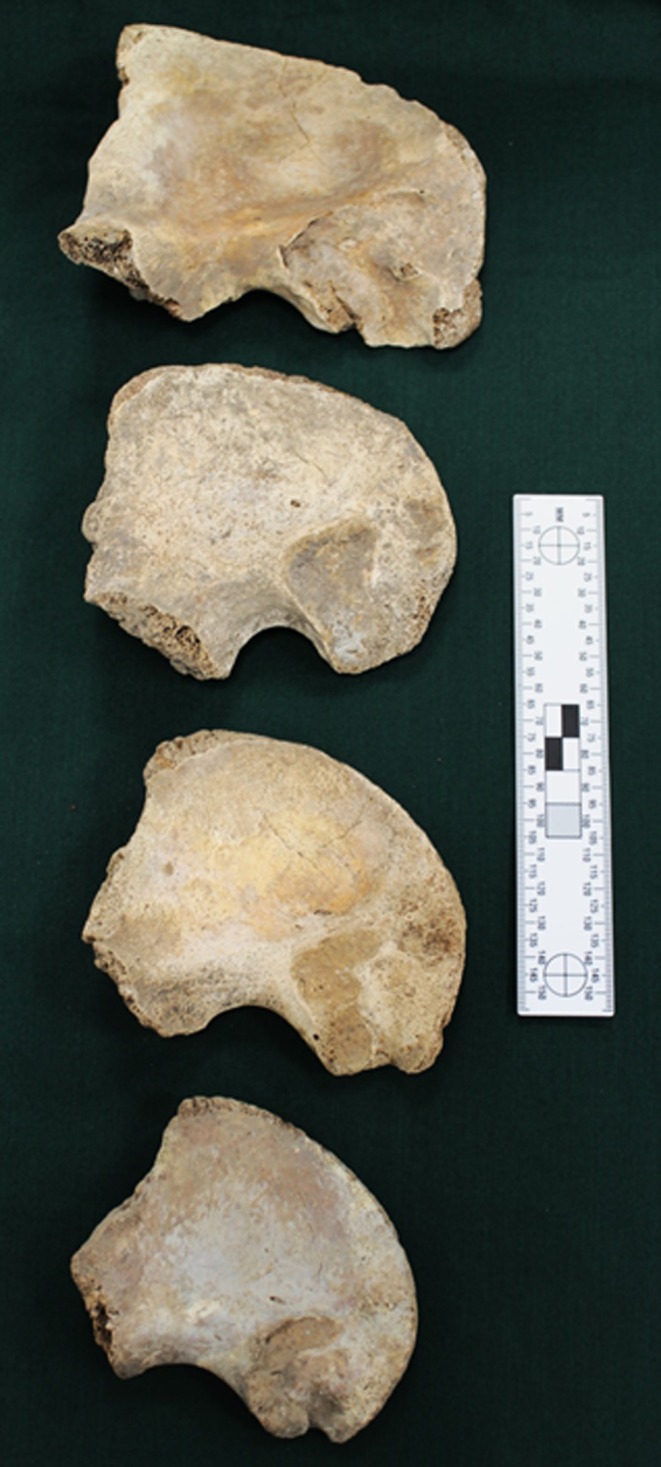
Four human non‐adult ilia representing different developmental stages from the same archaeological context (Context C).

**FIGURE 13:**
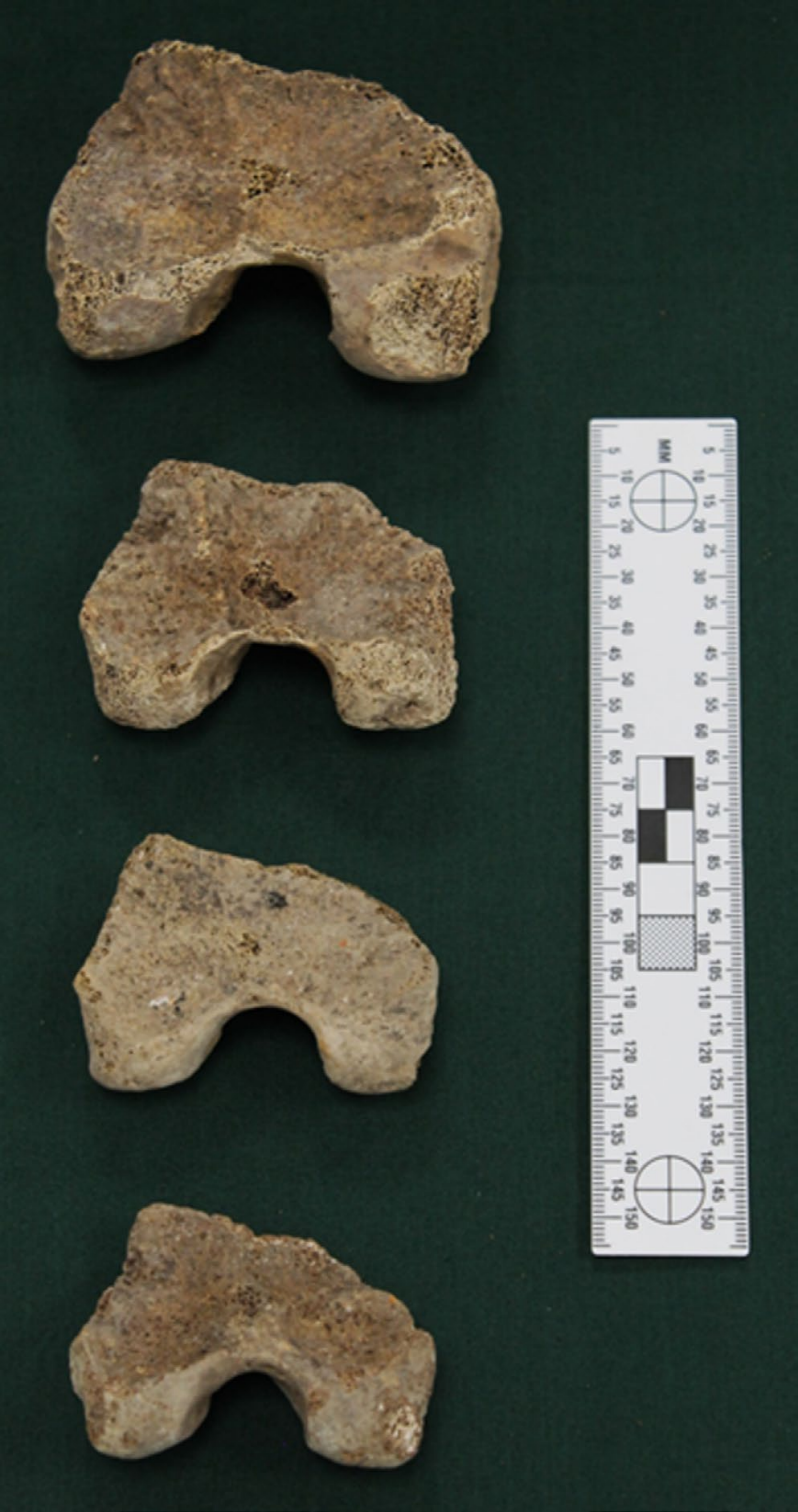
Four human non‐adult distal femoral epiphyses representing different developmental stages from Context D.

The age distribution of the non‐adult remains is unlikely to reflect simple mortality patterns alone. Infants and very young children are comparatively under‐represented, whereas older children and adolescents are more frequently represented. This pattern is partly attributable to preservation bias but likely also reflects pedagogical selection. Foetal and infant bodies were particularly valued for demonstrating developmental anatomy and were often preserved and retained within teaching collections rather than routinely discarded (Dittmar & Mitchell, 2016). In contrast, older children and adolescents may have been more likely to enter the routine cycle of dissection and eventual disposal. The recovery of four non‐adult ilia representing different developmental stages from the same archaeological context (Context C; see Figure 12), together with a separate group of four non‐adult distal femoral epiphyses from Context D (see Figure 13), is consistent with this interpretation. This pattern of grouped remains is unlikely to represent random dissection debris and more plausibly reflects the discard of a curated comparative teaching set used to illustrate growth‐ and age‐related skeletal variation (Dittmar & Mitchell, 2016).

Overall, the non‐adult remains provide insight not only into the presence of child bodies within the anatomy school, but also into their specific pedagogical roles. As with the adult assemblage, their representation appears to have been shaped by both biological factors and institutional practices of selection, retention and disposal, highlighting the structured and didactic use of human material in nineteenth‐century anatomical education.

### 4.4 Pedagogical use of human remains and specimen curation

The anatomical distribution of skeletal elements provides insight into the organisation of anatomical teaching during the nineteenth century. Pelvic elements, femora and cranial fragments are particularly well represented, whereas vertebrae and smaller elements of the limbs are comparatively scarce. This uneven distribution indicates selective retention and discard rather than the disposal of intact bodies.

One explanation for this uneven distribution lies in the logistical constraints of anatomical teaching. At this time, shortages of anatomical material frequently required bodies to be divided into discrete anatomical regions so that multiple students could dissect simultaneously, a practice often termed ‘body sharing’ (Richardson, 2001). Under such conditions, certain anatomical regions may have been circulated as discrete ‘units’ within the teaching environment and been discarded as such after use, while others, particularly non‐duplicated elements, were retained for demonstration purposes or inclusion in reference or ‘museum’ collections.

The predominance of pelvic elements and femora may reflect their frequent use as discrete ‘units’, while the relative scarcity of smaller elements may indicate preferential retention within anatomical collections. Cranial fragments are particularly abundant and frequently exhibit chemical whitening and evidence of preparation. The skull occupies a central role in anatomical education and was commonly prepared for teaching and display. However, the skull is structurally fragile and highly susceptible to damage during repeated handling in teaching environments, which likely contributed to fragmentation and eventual discard.

Evidence of post‐mortem modification is limited, with only approximately 3% of fragments displaying cut or saw marks. Although this proportion appears low for material derived from an anatomy school, it is consistent with experimental studies showing that skeletal modification during careful anatomical dissection is limited (Dittmar & Mitchell, 2015). Nineteenth‐century anatomical dissection practices primarily aimed to expose soft tissues carefully while preserving skeletal integrity for further study or display (Dittmar & Mitchell, 2016). Cut marks, therefore, tend to occur only where instruments repeatedly and forcefully contact bone surfaces (Greenfield, 1999; Shipman & Rose, 1983). Saw marks are largely confined to cranial elements and are most plausibly associated with craniotomy, while postcranial saw modifications may relate to the division of bodies for the purpose of ‘body sharing’ rather than dissection itself.

Additional evidence, including metal fittings, drill holes, ink and pencil markings and chemically whitened bone, indicates that part of the assemblage may derive from a curated anatomical teaching collection prepared with care. Approximately 20% of fragments exhibited whitening, the majority of which were cranial elements, suggesting chemical treatment, a preparation technique commonly used to clean and lighten skeletal material intended for anatomical demonstration or display. These observations support the interpretation that the assemblage represents the discard of teaching material that had undergone careful, intentional preparation and prolonged use before becoming obsolete.

The pathological profile is consistent with post‐medieval urban populations, with evidence of infectious disease, degenerative conditions and nutritional stress. The relative scarcity of rare or severe pathological or traumatic specimens may reflect selective retention of more distinctive or instructive specimens for teaching collections rather than their absence within the source population.

Taken together, the patterned distribution of skeletal elements, limited evidence of dissection‐related modification and clear indicators of careful preparation and curation indicate that the assemblage represents the final stage in the life cycle of anatomical teaching material. The remains appear to derive from specimens that were repeatedly used, curated and selectively retained before ultimately being discarded once they had lost pedagogical value. As such, the assemblage provides insight not only into dissection practices, but also into the organisation, reuse and eventual obsolescence of anatomical material within the nineteenth‐century teaching environment.

This interpretation challenges the implicit assumption that anatomy‐school deposits primarily represent the by‐products of dissection, suggesting instead that they may also derive from the structured life cycle of curated teaching material. At the same time, the available evidence suggests that the deposit was heterogeneous and likely contained both routinely dissected material and elements derived from prepared and curated teaching specimens.

### 4.5 Comparative anatomy and the role of animal material

The animal remains recovered from the quadrangle demonstrate that the assemblage reflects not only human dissection but also the practice of comparative anatomy, a central component of nineteenth‐century anatomical education (Waddington, 2012). The study of animal anatomy formed an important part of medical training, enabling students to examine anatomical structures, physiological systems and pathological processes across species. Animal material was also used to supplement human dissection, particularly when the supply of human remains for anatomical teaching was limited and it was employed in demonstrations of muscular, skeletal and visceral anatomy (Richardson, 2000).

This reliance on animal material must be understood within the broader context of nineteenth‐century anatomy teaching, in which demand for human bodies was high but supply remained constrained both before and after the Anatomy Act of 1832 (Richardson, 2000). Demand for anatomical material was considerable; Richardson (2000) estimates that more than 800 students were enrolled across London anatomy schools during the nineteenth century, with approximately 500 actively engaged in dissection. Public anatomical demonstrations also attracted audiences of between 100 and 300 attendees per session (Richardson, 2000; Waddington, 2000), further increasing the need for suitable teaching material. The use of animal specimens therefore not only supplemented human dissection but helped sustain large cohorts of students and the expanding demands of anatomical instruction.

The commingling of animal and human remains within the assemblage reflects the broader pedagogical environment of the UCL anatomy school, in which both human and animal bodies contributed to the production and transmission of anatomical knowledge.

The assemblage is dominated by domestic species, particularly cattle, sheep/goat and horse, with additional representation of pig, dog and smaller taxa. This composition suggests a pragmatic approach to teaching, focused on readily available and economically viable material rather than rarer taxa or more exotic species. Large mammals such as cattle provided robust skeletal elements and sizeable anatomical structures for demonstration, while medium‐sized animals such as sheep/goat and dog were well suited to whole‐body dissection.

Although some material may represent domestic or kitchen waste, several specimens exhibit modifications consistent with anatomical use, including sawn skulls, drilled elements and chemically whitened bone. These features indicate deliberate preparation and repeated handling consistent with teaching and display. The presence of butchery marks, particularly on cattle elements, suggests that some anatomical material may have been acquired as disarticulated ‘meat‐market’ cuts rather than deriving from whole carcasses and subsequently repurposed for anatomical instruction. In contrast, the comparatively low frequency of butchery marks on sheep/goat remains indicates that these animals may have been dissected as complete individuals. The non‐human assemblage is therefore best interpreted as a mixed deposit, in which comparative anatomical material was discarded alongside more routine refuse, including a quantity of oyster shells, which were a cheap and widely consumed food among UCL students during this period (Brewis & Blaxland, 2026). Although some faunal material may derive from butchery or general refuse, the assemblage as a whole cannot be explained solely in these terms.

The species profile is also informative regarding the organisation of anatomical teaching at UCL and likely reflects the influence of particular individuals. Comparative anatomy at UCL was shaped in part by Robert Edmond Grant (1793–1874) and William Youatt (1776–1847), who represent the most plausible contributors to this material. Both held roles in comparative anatomy at UCL during the institution's formative period of anatomical teaching. Grant served as lecturer and curator at UCL from 1826 until his death in 1874, while Youatt held a lectureship in comparative anatomy between approximately 1830 and 1835 (Lee, 1900). Contemporary evidence indicates that both men were actively advertising their lectures to medical students in The Lancet, where courses in comparative and veterinary anatomy were described as being ‘*illustrated by an extensive veterinary museum*’ (*Lancet*, 1832, p. 9). This emphasis on demonstrative teaching aligns with the composition of the assemblage. Grant's work encompassed a wide zoological range, including birds, fish, amphibians, invertebrates and small mammals, as well as occasional exotic specimens such as marine invertebrates and large mammals, including elephants and tigers (Grant, 1828). In contrast, Youatt focused primarily on domesticated species such as cattle, sheep and horses (Youatt, 1832).

The predominance of large domestic animals within the assemblage may align more closely with Youatt's pedagogical focus. However, this does not exclude Grant's influence; rather, it suggests that smaller or more unusual specimens may have preferentially been retained within anatomical collections, while more routine material entered the discard assemblage. Some of these curated specimens likely survive today within the UCL Grant Museum of Zoology, reflecting longer term curatorial practices of retention and display. The animal assemblage therefore reflects both teaching practice and selective curation and may be shaped by the differing pedagogical approaches and research interests of Grant and Youatt, although attributing specimens to either individual is not possible. Their eventual discard may relate to later phases of reorganisation within the anatomy department, in which older teaching material, potentially accumulated during or shortly after their tenure, was cleared out once it had lost pedagogical value.

**FIGURE 14:**
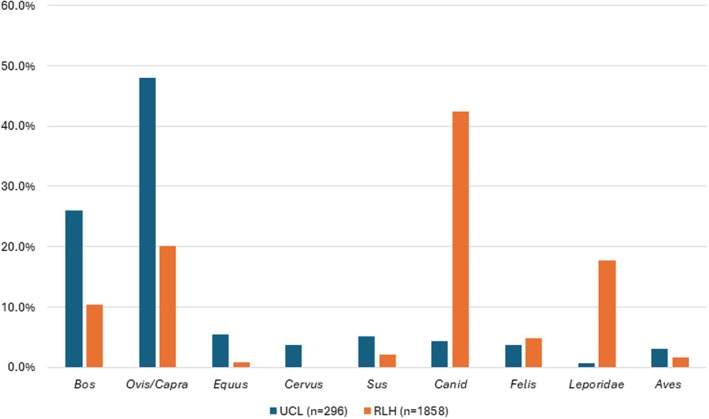
Relative distribution of species found at UCL compared with the same species from Royal London Hospital (RLH).

Comparison with other excavated assemblages further supports the interpretation that the UCL animal material relates primarily to anatomical teaching rather than to domestic food waste. The animal assemblage from the Royal London Hospital (RHL), for example, provides useful contextual insight (Morris, 2014), although important differences between the sites must be acknowledged. The RLH animal assemblage contains substantial quantities of domestic refuse associated with hospital diet, and many of the recovered remains are partially or fully articulated. Such deposits can inflate fragment counts and skew species representation when compared with a context such as the UCL quadrangle, which appears to derive from a more selective depositional process. To facilitate comparison, the relative distribution of overlapping species at both sites was collated (see Figure 14).

Domesticated species such as cattle and sheep/goat are considerably more abundant at UCL, whereas dogs and rabbits dominate the RLH assemblage. Both sites display relatively low frequencies of horse and pig remains. The lower representation of cattle and sheep/goat at RLH is particularly notable given their prominence in the hospital diet (Morris, 2014). Their relative abundance at UCL is consistent with the interpretation that these remains primarily represent anatomical teaching material, particularly the kind associated with Youatt, rather than food waste. In contrast, the RLH assemblage includes several exotic species, such as tortoise, monkey and guinea pig, which are absent from the UCL deposit. This difference may reflect curatorial practices at UCL, whereby unusual or rare specimens were preferentially retained for teaching or museum display rather than discarded. The low frequency of pig remains at both sites is also notable. Although anatomically similar to humans, pigs were found to be less suitable for dissection due to their high adipose content, which complicates dissection, obscures delicate anatomical structures and leads to rapid decomposition. Contemporary accounts suggest that leaner animals, such as sheep and dogs, were often preferred for dissection (Guerrini, 2015). Sheep brains, in particular, were used in comparative neuroanatomy teaching because they were inexpensive, readily available as offal and anatomically comparable to human brains (Shepherd, 2010).

The UCL animal assemblage reflects the practical realities of comparative anatomical teaching in the nineteenth century, in which domesticated animals appear to have been used to supplement human dissection, enabling students to observe anatomical structures while reducing reliance on scarce human bodies and supporting growing student cohorts within an expanding medical curriculum. While the identified species clearly indicate that these animals were used within the anatomy department at some stage, their commingling with the human material also suggests shared pathways of use, curation and disposal within the same teaching environment. As with the human material, these remains formed part of a broader pedagogical and curatorial system involving acquisition, preparation, repeated use, selective retention and eventual discard. The assemblage therefore provides rare material evidence for the integrated use of human and animal bodies in anatomical education and highlights the role of comparative approaches in the production and transmission of anatomical knowledge at UCL.

### 4.6 Ethical and legal perspectives on historic anatomical remains

The UCL assemblage raises important ethical and legal questions concerning the acquisition, use and treatment of human remains in anatomical education. The individuals represented within the assemblage almost certainly entered the anatomy school through the systems of body procurement established following the Anatomy Act of 1832. Although the Act addressed the chronic shortage of bodies available for anatomical teaching, it did so largely by granting anatomists access to the unclaimed dead from workhouses, hospitals and other public institutions (Richardson, 2000). As a consequence, the burden of supplying anatomical material fell disproportionately upon the poor and socially marginalised, many of whom would not have provided explicit consent for dissection in the manner expected today (Hurren, 2012; Richardson, 1987). Understanding this history helps illuminate contemporary ethical issues in anatomy and medicine and underscores the importance of integrating ethics, professionalism and respect for human remains within anatomy education (Hildebrandt, 2019; Leeper et al., 2024; Stephens et al., 2019). The assemblage therefore reflects not only the development of anatomical science but also the social inequalities embedded within nineteenth‐century systems of anatomical education.

Modern anatomical practice operates within a fundamentally different ethical framework. Contemporary body donation programmes in the United Kingdom are founded upon the fundamental principle of informed consent, with individuals voluntarily choosing to donate their bodies for education and research in accordance with the Human Tissue Act 2004 and the Human Tissue Authority's Codes of Practice (Human Tissue Authority, 2025). The contrast between modern donation programmes and the historical acquisition of unclaimed bodies highlights the significant ethical transformation that has occurred in attitudes towards bodily autonomy, consent and the treatment of the dead. The remains recovered from the UCL quadrangle therefore occupy a complex position, representing individuals who contributed to the advancement of anatomical knowledge but whose participation in that process was unlikely to have been voluntary.

The discovery of these remains also raises questions regarding their current status and future use. Although archaeological human remains occupy a different legal category from modern donated bodies, they continue to warrant respectful treatment and careful stewardship. Their scientific and historical value lies not only in the information they provide regarding nineteenth‐century anatomical education but also in their capacity to illuminate the lived experiences of the individuals whose bodies entered the anatomy school. Research, teaching and, where appropriate, public display, therefore require a balance between educational benefit and ethical responsibility. In this respect, the UCL assemblage serves as a reminder that the history of anatomy is not solely a history of scientific progress but also one of human lives, social circumstance and changing ethical values. The remains recovered from the quadrangle are therefore significant not only as archaeological evidence of anatomical practice but also as individuals whose bodies formed part of the complex historical relationship between medicine, education and society.

### 4.7 Contemporary legal and professional frameworks

The legal and ethical status of historic anatomical remains differs substantially from that of contemporary donated bodies. The legal framework governing anatomical material in the United Kingdom has evolved substantially since the Anatomy Act 1832. While the Anatomy Act sought to regulate the supply of bodies for anatomical teaching, subsequent legislation increasingly emphasised consent and individual autonomy. The Human Tissue Act 1961 introduced provisions relating to the use of bodies and body parts for medical purposes, while the Human Tissue Act 2004 established informed consent as the central principle governing the storage and use of human tissue and bodies for education and research. In the United Kingdom, the use of bodies donated for anatomical examination is regulated by the Human Tissue Authority under the provisions of the Human Tissue Act 2004. However, archaeological human remains recovered from historic contexts are generally subject to a different regulatory framework and are commonly managed through a combination of burial legislation, museum standards, professional guidance and institutional governance. Current guidance issued by organisations such as the British Association for Biological Anthropology and Osteoarchaeology, the Chartered Institute for Archaeologists, Historic England and the Department for Culture, Media and Sport emphasises the need for respectful treatment, clear justification for research, appropriate storage and careful consideration of public display. Within this framework, the scientific, educational and historical value of human remains must be balanced against wider ethical responsibilities towards the individuals represented. The UCL assemblage is therefore significant not only as archaeological evidence of nineteenth‐century anatomical practice but also as a collection of human remains requiring ongoing stewardship in accordance with contemporary professional and ethical standards. While these remains can continue to contribute to research, teaching and public understanding, their use should be guided by principles of dignity, respect, proportionality and transparency.

### 4.8 Disposal and the lifecycle of anatomical material

The archaeological context of the assemblage raises important questions regarding the final disposal of anatomical material. The deposit's location within the highly prominent front quadrangle, together with the absence of documentary evidence in surviving UCL records, suggests it was unlikely that the quadrangle would have served as a routine burial site for dissected remains. Instead, the evidence points to a single or limited episode of disposal involving anatomical material, including carefully prepared specimens from a curated collection, that was no longer considered useful for teaching.

Under the Anatomy Act of 1832, dissected bodies were required to receive a ‘decent burial’, although the legislation did not define the precise form such a burial should take nor require interment in consecrated ground (Richardson, 1987). In practice, this generally meant burial in a cemetery or burial ground, typically out of public view and often accompanied by a record of interment (Richardson, 1987), rather than disposal within an institutional courtyard. No records of interment have been identified in the surviving UCL archives.

The assemblage is therefore most plausibly interpreted as a deposit resulting from the informal disposal, rather than formal burial, of anatomical material. The public and central location of the quadrangle further supports the interpretation that this was not an established or routine disposal site. Instead, the assemblage likely represents the clearance of anatomical teaching material, including specimens from a curated collection, that had undergone prolonged use and was no longer considered suitable for retention. The absence of documentary evidence for associated building or excavation works during this period further suggests that this disposal occurred opportunistically and without formal record.

In this respect, the assemblage provides important insight into the life cycle of anatomical material within nineteenth‐century medical institutions. Bodies used for anatomical teaching were acquired through institutional networks, dissected by students, selectively prepared as teaching specimens, retained for varying periods and ultimately discarded once their perceived pedagogical value diminished. The UCL quadrangle deposit represents the final stage of this process and provides rare archaeological evidence for the material practices that underpinned anatomical education.

## 5 CONCLUSION

The UCL quadrangle assemblage provides rare archaeological evidence for the material practices of nineteenth‐century anatomical education in London. The human remains suggest that anatomical teaching at UCL was sustained by the institutional systems of anatomical material supply formalised after the Anatomy Act of 1832, and that these systems disproportionately drew on socially and economically marginalised populations. The demographic profile, dominated by adults aged 30–50 years and showing an overall male:female ratio of approximately 2:1, together with the pathological evidence, is consistent with the incorporation of socially and economically marginalised populations into medical education through institutional networks, particularly the workhouse system. Although direct attribution to specific institutional sources is not possible, the observed patterns are consistent with these broader systems of acquisition. The presence of non‐adult remains further demonstrates that child bodies also formed part of anatomical teaching, probably in specific pedagogical contexts linked to growth and development.

At the same time, the assemblage shows that the quadrangle deposit did not simply comprise the final products of routine student dissection. The low frequency of cut marks, the patterned anatomical representation and the evidence for whitening, drilling, metal fittings and labelling all suggest that at least part of the assemblage derived from prepared and curated teaching material that had undergone repeated use before eventual discard. The associated animal remains extend this interpretation beyond human dissection alone. Their taxonomic composition, anatomical distribution and patterns of modification indicate that domesticated animals formed an important component of comparative anatomy teaching at UCL and were subject to similar processes of acquisition, preparation, use, selective retention and disposal.

The UCL assemblage demonstrates the value of archaeology in reconstructing the history of anatomy education by revealing aspects of anatomical practice rarely captured in documentary sources, including the practical realities of acquisition, preparation, curation and the ultimate disposal of both human and animal material in the service of medical education. The assemblage therefore provides more than evidence of dissection; it also reveals the broader lifecycle of anatomical material within the medical school and the practical organisation of both human and comparative anatomy. Beyond documenting anatomical practice, the assemblage highlights the ethical complexities of human remains acquisition, use and disposal, and invites reflection on changing standards of consent and stewardship. It therefore provides a rare archaeological perspective on both the practical and ethical dimensions of nineteenth‐century anatomical education.

As University College London marks its bicentenary in 2026, the quadrangle assemblage offers a unique opportunity to reflect on the material history of dissection at UCL and on the practices through which anatomical knowledge was produced and transmitted within one of Britain's earliest modern universities. While Bentham's ‘Auto‐Icon’ symbolises the intellectual ideals surrounding the use of the body in science, the remains recovered from beneath the quadrangle represent the largely anonymous human and animal bodies that sustained the everyday practice of anatomical teaching and the advancement of anatomical science. The assemblage therefore forms a rare material link between the intellectual history of anatomy and its lived realities, foregrounding the often‐overlooked bodies upon which two centuries of anatomical education at UCL were built.

## AUTHOR CONTRIBUTIONS

Wendy Birch: Conceptualisation, investigation, analysis, interpretation, writing, review and editing. Tania Kausmally: Investigation, analysis, data curation, interpretation, writing, review and editing.

## FUNDING INFORMATION

This research received no specific grant from any funding agency in the public, commercial or not‐for‐profit sectors.

## CONFLICT OF INTEREST STATEMENT

The authors declare no conflicts of interest.

## ETHICS STATEMENT

This study is based on archaeological human and animal remains recovered during a rescue excavation at University College London. Analysis was undertaken with institutional permission and in accordance with relevant professional standards for the study of archaeological human remains. Formal research ethics approval was not required.

## Data Availability

The data that support the findings of this study are available from the corresponding author upon reasonable request.
